# Statistical Machine Learning for Human Behaviour Analysis

**DOI:** 10.3390/e22050530

**Published:** 2020-05-07

**Authors:** Thomas B. Moeslund, Sergio Escalera, Gholamreza Anbarjafari, Kamal Nasrollahi, Jun Wan

**Affiliations:** 1 Visual Analysis of People Laboratory, Aalborg University, 9000 Aalborg, Denmark; tbm@create.aau.dk; 2 Computer Vision Centre, Universitat Autònoma de Barcelona, Bellaterra (Cerdanyola), 08193 Barcelona, Spain; sergio@maia.ub.es; 3 Department of Mathematics and Informatics, Universitat de Barcelona, 08007 Barcelona, Spain; 4 iCV Lab, Institute of Technology, University of Tartu, 50411 Tartu, Estonia; shb@ut.ee; 5 Department of Electrical and Electronic Engineering, Hasan Kalyoncu University, 27900 Gaziantep, Turkey; 6 PwC Finland, Itämerentori 2, 00100 Helsinki, Finland; 7 Research Department of Milestone Systems A/S, 2605 Copenhagen, Denmark; 8 National Laboratory of Pattern Recognition (NLPR), Institute of Automation, Chinese Academy of Sciences, Beijing 100190, China; jun.wan@ia.ac.cn

**Keywords:** action recognition, emotion recognition, privacy-aware

Human behaviour analysis has introduced several challenges in various fields, such as applied information theory, affective computing, robotics, biometrics and pattern recognition. This Special Issue focused on novel vision-based approaches, mainly related to computer vision and machine learning, for the automatic analysis of human behaviour. We solicited submissions on the following topics: information theory-based pattern classification, biometric recognition, multimodal human analysis, low resolution human activity analysis, face analysis, abnormal behaviour analysis, unsupervised human analysis scenarios, 3D/4D human pose and shape estimation, human analysis in virtual/augmented reality, affective computing, social signal processing, personality computing, activity recognition, human tracking in the wild, and application of information-theoretic concepts for human behaviour analysis. In the end, 15 papers were accepted for this special issue [1,2,3,4,5,6,7,8,9,10,11,12,13,14,15]. These papers, that are reviewed in this editorial, analyse human behaviour from the aforementioned perspectives, defining in most of the cases the state of the art in their corresponding field.

Most of the included papers are application-based systems, while [15] focuses on the understanding and interpretation of a classification model, which is an important factor for the classifier’s credibility. Given a set of categorical data, [15] utilizes multi-objective optimization algorithms, like ENORA and NSGA-II, to produce rule-based classification models that are easy to interpret. Performance of the classifier and its number of rules are optimized during the learning, where the first one is obviously expected to be maximized while the second one is expected to be minimized. Testing on public databases, using 10-fold cross-validation, shows the superiority of the proposed method against classifiers that are generated using other previously published methods like PART, JRip, OneR and ZeroR.

Two published papers ([1,9]) have privacy as their main concern, while they develop their respective systems for biometrics recognition and action recognition. Reference [1] has considered a privacy-aware biometrics system. The idea is that the identity of the users should not be readily revealed from their biometrics, like facial images. Therefore, they have collected a database of foot and hand traits of users while opening a door to grant or deny access, while [9] develops a privacy-aware method for action recognition using recurrent neural networks. The system accumulates reflections of light pulses omitted by a laser, using a single-pixel hybrid photodetector. This includes information about the distance of the objects to the capturing device and their shapes.

Multimodality (RGB-depth) is covered in [14] for sign language recognition; while in [11], multiple domains (spatial and frequency) are used for saliency detection. Reference [14] has applied restricted Boltzmann machine (RBM)s to develop a system for sign language recognition from a given single image, in two modalities of RGB and depth. Two RBMs are designed to process the images coming from the two deployed modalities, while a third RBM fuses the results of the first two RBMs. The inputs to the first two RBMs are hand images that are detected by a convolutional neural network (CNN). The experimental results reported in [14] on two public databases show the state-of-the-art performance of the proposed system. Reference [11] proposes a multi-domain (spatial and frequency)-based system for salient object detection in foggy images. The frequency domain saliency map is extracted using the amplitude spectrum, while the spatial domain saliency map is calculated using the contrast of the local and global super-pixels. These different domain maps are fused using a discrete stationary wavelet transform (DSWT) and are then refined using an encoder-decoder model to pronounce the salient objects. Experimental results on public databases and comparison with state-of-the-art similar methods show the better performance of this system. 

Four papers in this special issue have covered action recognition [6,9,12,13]. Reference [12] has proposed a system for toe-off detection using a regular camera. The system extracts the differences between consecutive frames to build silhouettes difference maps, that are then fed into a CNN for feature extraction and classification. Different types of maps are developed and tested in this paper. The experimental results reported in [12] on public databases show state-of-the-art performance. Reference [6] proposes a system for individuals and then crowd condition monitoring and prediction. Individuals participating in this study are grouped into crowds based on their physical locations extracted using GPS on their smartphones. Then, an enhanced context-aware framework using an algorithm for feature selection is used to extract statistical-based time-frequency domain features. Reference [13] focuses on utilizing recurring concepts using adaptive random forests to develop a system that can cope with drastically changing behaviours in dynamic environments, like financial markets. The proposed system is an ensemble-based classifier comprised of trees that are either active or inactive. The inactive ones keep a history of market operators’ reactions in previously recorded similar situations, while either an inactive tree or a background tree that has recently been trained replaces the active ones, as a reaction to drift. 

In terms of face analysis, in [10] a system is proposed for detecting fuzziness tendencies and utilizing these to design human-machine interfaces. This is motivated by the fact that humans tend to pay more attention to sections of information with fuzziness, which are sections with greater mental entropy. The work of [4] proposes a conditional random field-based system for segmentation of facial images into six facial parts. These are then converted into probability maps, which are used as feature maps for a random decision forest that estimates head-pose, age, and gender. 

The method introduced in [3] uses singular value decomposition for removing background of fingerprint images. Then, it finds fingerprints’ boundaries and applies an adaptive algorithm based on wavelets extrema and Henry system to detect singular points, which are widely used in applications related to fingerprint, like registration, orientation detection, fingerprint classification, and identification systems. 

Three papers have covered emotion recognition, one from body movements [5], and two from speech signals [2,7]. In [2] a committee of classifiers has been applied to a pool of descriptors extracting features from speech signals. Then, it is used as a voting scheme on the classifiers’ outputs to get to a conclusion about the emotional status from the used speech signals. The paper in [2] shows that the committee of classifiers outperforms the single individual classifiers in the committee. The system proposed in [7] builds 3D tensors of spectrogram frames that are obtained by extracting 88-dimentional feature vectors from speech signals. These tensors are then used for building a 3D convolutional neural network that is employed for emotion recognition. The system has produced state-of-the-art results on three public databases. The emotional recognition system of [5] does not use facial images or speech signals, but body movements, which are captured by Microsoft Kinect v2 under eight different emotional states. The affective movements are represented by extracting and tracking location and orientation of body joints over time. Experimental results, using different deep learning-based methods, show the state-of-the-art performance of this system. 

Finally, two databases have been introduced in this special issue, one for biometric recognition [1] and one for detecting sleeping issues and fatigue [8], the later containing a database of patients suffering from Fibromyalgia, which is a situation resulting in muscle pain and tenderness, accompanied by few other signs including sleep, memory, and mood disorders. It uses similarity functions with configurable convexity or concavity to build a classifier on this collected database in order to predict extreme cases of sleeping issues and fatigue.
